# Technical note: novel delivery methods for an enterotoxigenic Escherichia coli infection model in MUC4-locus sequenced weaner pigs^1^

**DOI:** 10.1093/jas/skz303

**Published:** 2019-09-23

**Authors:** Samantha O Sterndale, David W Miller, Josie P Mansfield, Jae C Kim, Mark O’Dea, John R Pluske

**Affiliations:** 1 Agricultural Sciences, College of Science, Health, Engineering and Education, Murdoch University, Murdoch, WA, Australia; 2 AB Vista Asia Pte. Ltd., The Mezzo, Whampoa, Singapore; 3 Antimicrobial Resistance and Infectious Disease Laboratory, Murdoch University, Murdoch, WA, Australia

**Keywords:** diarrhea, enterotoxigenic *Escherichia coli*, infection, MUC4, weaner pigs

## Abstract

An infection model with enterotoxigenic *Escherichia coli* (**ETEC**) harboring the F4 fimbriae can be used to assess the impacts that various challenges associated with weaning (e.g., dietary, psychological, environmental) have on the expression of postweaning diarrhea. The objective of this study was to develop a novel inoculation method for administering an ETEC culture that would induce a higher proportion of ETEC-F4 diarrhea, in pigs that genetically showed ETEC-F4 susceptibility or resistance. The study was designed as a factorial arrangement of treatments with the factors being 1) partially susceptible or resistant to ETEC-F4 based on genetic testing, and 2) 4 challenge treatments, being a) a conventional liquid broth method using a drenching gun [Positive control (**PC**)], b) a Syringe method, c) a Capsule method, and d) Negative control [pigs not challenged (**NC**)]. At 21 ± 3 d of age (mean ± SEM), 48 male castrate pigs (Large White × Landrace) weighing approximately 7.0 ± 1.18 kg were allocated to 4 treatment groups in 2 replicate pens (6 pigs per pen). Initial ETEC-F4 susceptibility was based on a DNA marker test and each treatment group had 9 partially susceptible and 3 resistant pigs. On days 7 and 8 after weaning, pigs were challenged with ETEC (serotype O149:K88; toxins LT1, ST1, ST2, and EAST). On each inoculation day the PC pigs were orally dosed with 9 mL 7.12 × 10^9^ colony-forming unit (**CFU**), the Syringe pigs with 0.8 mL 6.72 × 10^9^ CFU, the Capsule pigs were orally administered 2 capsules containing 0.8 mL 3.28 × 10^9^ CFU, and the NC pigs 1 mL of phosphate-buffered saline (**PBS**) solution. Approximately 72 h after infection, 44, 22, 78, and 0% of partially susceptible pigs in the PC, the Syringe, the Capsule, and the NC group had developed ETEC-F4 diarrhea (*P =* 0.007). Partially susceptible pigs had a higher diarrhea index (**DI**) compared to resistant pigs (31.5 vs. 4.8, *P* < 0.001). The NC group had a lower DI compared to the PC and Capsule pigs (3.9, 38.1, and 40.3, respectively, *P <* 0.005). Following infection, genetically resistant pigs in the Capsule group had a DI of zero and the partially susceptible pigs had a DI of 55.6 (*P* = 0.014). This study showed that genetically screening pigs and using a Capsule to deliver ETEC-F4 can increase cases of diarrhea and the efficiency of the challenge model. Taken together, these methods have the potential to reduce the number of pigs needed in future experimental infection studies.

## Introduction

An enterotoxigenic *Escherichia coli* F4 (**ETEC-F4**) infection model frequently has been used to mimic the impacts that various challenges associated with weaning (e.g., dietary, psychological, environmental) have on the expression of ETEC-F4 diarrhea. Conventionally, in this system pigs are dosed orally with a liquid broth culture of ETEC-F4 at some time point(s) after weaning, with diarrhea subsequently monitored. However, due to the generally high volume given, low palatability, stress of the procedure, and the time taken, there is often spillage and (or) aspiration of ETEC into the lungs (Kim et al., 2014; Luise et al., 2019). This cannot only lower the prevalence of diarrhea, but also present a potential welfare issue. Another factor that can influence diarrhea is the pigs’ genetic susceptibility to ETEC-F4. Pigs identified as susceptible have the appropriate F4 receptors in the small intestine allowing the ETEC to adhere to enterocytes, where they can release toxins that cause diarrhea to develop. Sequencing of DNA has identified the Mucin 4 (**MUC4**) gene on chromosome 13 (Fontanesi et al., 2012) where a single nucleotide polymorphism (DQ848681:g.8227C>G) called the *XbaI* polymorphism is located, as a marker for susceptibility. A Polymerase Chain Reaction-Restriction Fragment Length Polymorphism (**PCR-RFLP**) assay has been used to identify fully susceptible, partially susceptible, and resistant pigs. However, further investigation comparing the PCR-RFLP to Next-Generation DNA sequencing and subsequent diarrhea suggests that partially susceptible and resistant pigs are not significantly different in their MUC4 C:G allele percentage (Sterndale et al., 2019), inferring that other factors such as delivery method may play an important role in ETEC challenge models.

Therefore, the aim of this study was to determine if a more concentrated and lower volume of ETEC can illicit ETEC-F4 diarrhea. Furthermore, it was hypothesized that partially susceptible pigs will develop more ETEC-F4 diarrhea and shed more fecal ETEC than resistant pigs when experimentally challenged with the new Capsule delivery method.

## Materials and Methods

### Animals, Experimental Design, and Housing

This experiment was approved by the Animal Ethics Committee of Murdoch University (R2864/16). Bristles and the attached follicles were collected from 48 piglets 1 week before weaning (14 ± 3 d of age; mean ± SEM). This was done by gently restraining the piglets, plucking 10 to 20 bristles including the follicle, and placing the sample into sterile tubes on ice. Utensils and gloves were sanitized between each piglet to ensure no cross contamination occurred.

The study was designed as a factorial arrangement of treatments with the factors being 1) MUC4+ or MUC4−, and 2) 4 challenge treatments, being a) a conventional ETEC dosing method via a drench gun [Positive control (**PC**); see below], b) a Syringe method (explained later), c) a Capsule method (explained later), and d) Negative control [pigs not challenged (**NC**)]. All pigs were fed ad libitum the same diet of commercially available (Farmyard Pig Weaner; Weston Milling Animal Nutrition, Western Australia, Australia) weaner pellets (specifications as supplied by manufacturer: 20% crude protein, 5% fat, 1.2% SID lysine, 5% crude fiber, 0.85% Ca, and 0.4% salt).

At day 21 ± 3 of age (mean ± SEM), 48 male castrate pigs (Large White × Landrace) weighing approximately 7.0 ± 1.18 kg (mean ± SEM) were weaned from a commercial piggery in Western Australia. The pigs arrived at Murdoch University and were allocated to 4 different treatment groups in 2 replicate pens (6 pigs per pen) using a randomized block distribution depending on their MUC4+/− propensity (after Jensen et al., 2006), the sows’ parity, and weaning weight. Pigs were housed in 2 different rooms at a temperature of 28.0 ± 1.0 °C (mean ± SD) in pens of metal construction with plastic floors, allowing at least 0.41 m^2^ per pig. Pigs in the NC group (2 pens) were housed in a separate room to the other 3 treatment groups to avoid ETEC contamination. The pens were fitted with a nipple drinker, a 5-space feeder, and plastic bottles for enrichment purposes. The experiment lasted 21 d.

### DNA Marker-Based Test

To determine the absence or presence of the MUC4 allele, a PCR-RFLP assay was completed on 25 ng of genomic DNA (collected as per Sterndale et al., 2019) in a total volume of 25 μL, using 5 μL MyTaq Red Reaction buffer, 0.5 units of MyTaq HS DNA polymerase (Bioline, New South Wales, Australia), and 0.4 μM of each MUC4 primer: 5′-GTCCCTTGGGTGAGAGGTTA/5′-CACTCTGCCGTTCTCTTTCC. Thermocycling was performed as described by Jensen et al. (2006). Restriction enzyme digest with *XbaI* (Promega, Wisconsin, USA) to identify the polymorphism on 5 μL PCR product was completed overnight and then run on a 2% agarose gel containing GelRed (Biotium, California, USA) using 100-bp Gene ruler (Thermo Fisher Scientific, Massachusetts, USA) with electrophoresis for 120 min at 80 volts. Bands were visualized by BioRad GelDoc (Life Science, California, USA). Pigs were classified as either fully resistant if the allele was not digested by the enzyme and produced only a single band at 367 bp, fully susceptible if the allele was digested by *XbaI* into 2 bands at 151 and 216 bp, and partially susceptible if all 3 bands were present at 367, 216, and 151 bp.

### DNA Sequencing

To confirm absence or presence of the MUC4 mutation, Sanger sequencing was completed retrospectively on all the DNA samples to further analyze and/or confirm the genotype (MUC4+/−) of the pigs. For identification of susceptible or resistant pigs, the DNA reads were aligned to reference sequence DQ848681. Pigs were classified as resistant (**MUC4**−) if a C nucleotide was present at the *XbaI* polymorphism site and susceptible (**MUC4+**) in case of a G nucleotide (Sterndale et al., 2019).

### Infection With ETEC

On days 7 and 8 after weaning pigs were challenged with ETEC (serotype O149:K88; toxins LT1, ST1, ST2, and EAST). Briefly, an aliquot from stock ETEC stored at −80 °C was grown on a Tryptic Soy Agar (**TSA**) plate containing 5% defibrinated sheep blood (Thermo Scientific, Thebarton, Australia) overnight at 37 °C. A single colony with clear hemolysis was selected and added to 20 mL of sterile Tryptic Soy Broth (**TSB**) (Bacto TSB; Becton, Dickinson and Company, Maryland, USA) and incubated in a water bath overnight at 23 °C. The culture was centrifuged at 2,000 × *g* for 15 min, the supernatant discarded, and the pellet resuspended in 20 mL fresh TSB. From this suspension 4 mL was added to 400 mL of TSB and further incubated for 3.5 h at 37 °C with orbital shaking at 120 rpm. The culture was centrifuged at 2,000 × *g* for 15 min, the supernatant discarded, and the pellets resuspended in fresh cold PBS. An aliquot was taken to measure the concentration of bacteria.

For the PC group, the culture was kept on ice and pigs were orally dosed with approximately 9 mL of 7.12 × 10^9^ colony-forming units (**CFU**) on days 7 and 8 after weaning. Oral administration of ETEC was performed by manually restraining the pig, holding the head up with its mouth open, and administering the inoculum via a 6-mL drenching gun (Prima Tech, Neogen, USA). This procedure took approximately 60 s per pig partly due to having to load 4.5 mL inoculum into the drench gun twice. This was to allow the pig time to swallow the first 4.5 mL dose before administering the second. It was observed, though, that in approximately 66% of the pigs, the full dose was not swallowed because regurgitation of the culture occurred.

For the Syringe group, the culture was approximately 10 times as concentrated and pigs were therefore dosed with 0.8 mL of 6.72 × 10^9^ CFU. Oral administration of ETEC was performed by mildly restraining the pig and administering the inoculum via a 3-mL sterile syringe. This procedure took approximately 10 s per pig.

The culture used for the Capsule group was the same as the Syringe treatment group, but instead 0.4 mL was pipetted into gelatin capsules (size 1; 14.9 mm length) and snap-frozen on dry ice before storing at −20 °C for 24 to 72 h. Pigs in the Capsule treatment group were orally administered 2 gelatin capsules with each containing 1.64 × 10^9^ CFU, for a total dose of 3.28 × 10^9^ CFU/d. Each capsule was given individually to the back of the tongue to ensure they were swallowed entire. This procedure took approximately 15 s; however, both capsules were swallowed for each pig inoculated.

On both of the ETEC challenge days, 2 capsules were thawed and an aliquot was used to determine the ETEC dose administered. Capsules were either stored 48 or 72 h at −20 °C, prior to inoculation, and bacteria viability did not decrease during this time. In total (2 d of inoculation), pigs in the PC group received 1.44 × 10^10^ CFU, Syringe pigs received 1.34 × 10^10^ CFU, and Capsule pigs 6.56 × 10^9^ CFU.

The NC pigs received 1 mL of PBS solution using a 3-mL syringe, and took approximately 10 s per pig.

### Fecal Consistency Score and ETEC-F4 Diarrhea

Fecal consistency was visually assessed for each pig daily for the 21 d of the study using a 4-point scale, as follows: score (1) firm, (2) soft, spreads slightly, (3) soft and loose, (4) watery liquid consistency. Pigs with a score 4 diarrhea for ≥2 consecutive days between days 7 and 14 were identified as having ETEC-F4 diarrhea. Diarrhea index (**DI**) was measured as the number of days the pig had a score 4 diarrhea, and expressed as the percentage of days with diarrhea over 8 d after weaning (i.e., between days 7 and 14).

### Fecal β-Hemolytic ETEC Shedding and Multilocus Sequence Typing

On days 0, 7, 8, 9, 10, 11, and 13 after weaning, fecal rectal swabs were taken to determine the shedding of ETEC. These swabs were plated on TSA with 5% defibrinated sheep blood (Thermo Scientific, Thebarton, Australia), incubated overnight at 37 °C and visually assessed the following day based on the presence of hemolytic colonies. The relative numbers of ETEC on the plates were scored 0–5, with 0 being no growth, 1 being growth only in the first streak section and 5 being the highest possible growth (Heo et al., 2009). Total fecal ETEC shedding score was calculated as the sum of all swab plate scores from days 0, 7, 8, 9,10, 11, and 13. Illumina next generation sequencing was undertaken on isolated beta-hemolytic colonies (*n* = 4) to determine the serotype of the ETEC as the causative agent of diarrhea (as per http://mlst.warwick.ac.uk/mlst/dbs/Ecoli). Sequencing data were uploaded to the Centre for Genomic Epidemiology (http://www.genomicepidemiology.org/) for determination of multilocus sequence typing (**MLST**).

### Blood Sampling and Analysis

Blood samples were collected from 2 partially susceptible pigs per pen (*n* = 16) before infection on day 7 and after infection on day 10. No resistant pigs were blood sampled. Samples were taken using vena cava puncture into a 9-mL lithium heparin tube and a 9-mL EDTA tube with a 20-gauge, 38-mm needle and vacutainer. Blood from the lithium heparin tubes was processed by centrifugation at 2,000 × *g* for 15 min to separate the plasma, which was later stored at −20 °C until analysis. The EDTA tubes were stored at room temperature until whole blood cell counts were completed on the same day using a Hemavet HV950 analyzer. Haptoglobin in plasma was determined using a method based on Comparative Hematology International (Eckersall et al., 1999), and plasma urea nitrogen (**PUN**) was analyzed using a Beckman Coulter/Olympus Reagent kit (OSR6134). The C-reactive protein (**CRP**) content was measured on plasma collected on days 7 and 10 after weaning using a commercially available ELISA kit (Cat. No. DY2648; R&D Systems, Minneapolis, USA).

### Statistical Analysis

Statistical analysis for fecal shedding, ETEC-F4 diarrhea, the DI, blood measures, and growth performance were completed using SPSS v. 24 (IBM SPSS, USA). Total ETEC shedding scores (swab scores) were analyzed using a generalized linear model comprising the ETEC-F4 susceptibility, 4 treatment groups (PC, Syringe, Capsule, and NC), and their interactions. The DI was abnormally distributed and transformation did not correct this. Therefore, raw means are presented and the significance between DI and the 4 treatment groups and DI and the ETEC-F4 susceptibility was tested using the nonparametric Kruskal–Wallis and Mann–Whitney *U*-tests, respectively. The interaction between DI, treatment, and ETEC-F4 susceptibility could not be analyzed due to the abnormal distribution. The percentage of pigs that developed ETEC-F4 diarrhea (score 4 for ≥2 consecutive days) between days 7 and 14 was analyzed using chi-square. All blood measures were analyzed using a generalized linear model, analyzing the effects of pre- and postinfection time points on the 4 treatment groups. Haptoglobin results were abnormally distributed and therefore log-transformed, and presented results are back-transformed. Significant differences were accepted at *P <* 0.05, and a trend was considered when 0.05 *< P <* 0.10.

## Results

As determined by the PCR-RFLP, each treatment group had 9 partially susceptible pigs and 3 resistant pigs. One pig in the PC group died during the study and another from the PC group was removed from analysis due to developing respiratory disease (as per veterinary diagnosis) following infection.

A significant difference was observed between treatments and ETEC-F4 diarrhea (*P =* 0.007), with 44, 22, 78, and 0% of partially susceptible pigs in the PC group, the Syringe group, the Capsule group, and the NC group developing ETEC-F4 diarrhea approximately 72 h after infection, respectively. Pigs determined to be resistant did not develop ETEC-F4 diarrhea within 72 h of infection irrespective of treatment groups.

**Figure 1.**
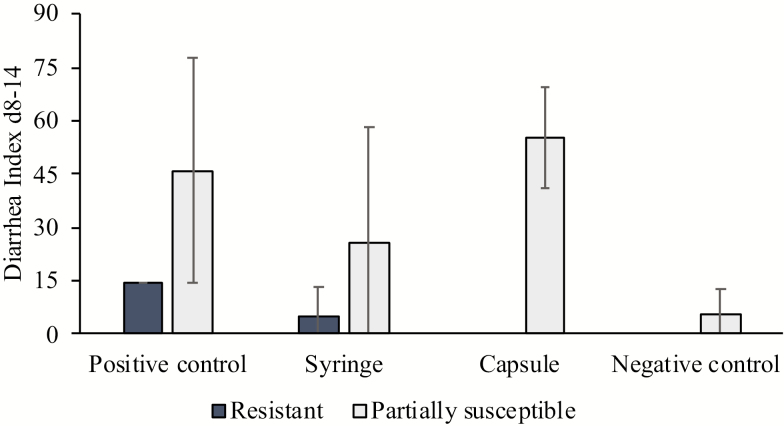
Effects of the 4 different treatment groups and enterotoxigenic *Escherichia coli* (ETEC)-F4 susceptibility (based on the DNA marker-based test) on the diarrhea index (DI) between days 8 and 14 after weaning. Data are presented as raw means (with SE).

Pigs determined to be partially susceptible had a higher DI compared to the resistant pigs (31.5 vs. 4.8, *P* < 0.001). For overall treatment effects, the NC group had a lower DI compared to the PC and Capsule treatment groups (3.9, 38.1, and 40.3, respectively, *P <* 0.005). Following infection, resistant pigs in the Capsule group had a DI of zero and partially susceptible pigs in the same group had a DI of 55.6 (*P* = 0.014; Fig. 1). There were no statistical differences between any other groups.

**Figure 2.**
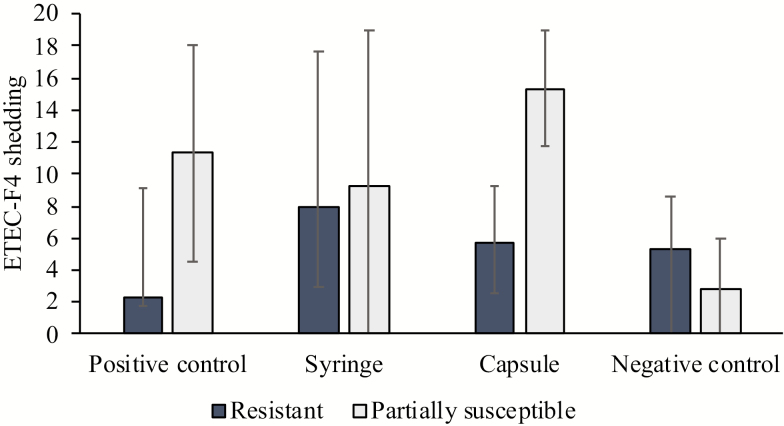
Effects of the 4 different treatment groups and enterotoxigenic *Escherichia coli* (ETEC)-F4 susceptibility (based on the DNA marker-based test) on total ETEC-F4 shedding. Total ETEC-F4 shedding score was determined by combining results from all swab plate scores from days 0, 7, 8, 9, 10, 11 and 13 after weaning. Data are presented as raw means (with SE).

Partially susceptible pigs shed more ETEC-F4 compared to resistant pigs (9.6 vs. 5.3, *P* = 0.02). Pigs in the Capsule group had a higher total ETEC-F4 shedding score compared to NC pigs (12.9 vs. 3.46, *P* = 0.013; Fig. 2), but there were no statistical differences between any other treatments. The MLST completed on fecal samples after infection confirmed the ETEC shed was the same serotype as the ETEC pigs were inoculated with.

White blood cell count, neutrophil, lymphocyte, PUN, and haptoglobin levels did not differ (*P* > 0.05) at day 7 or day 10 between the 4 treatment groups. The neutrophil to lymphocyte ratio was higher in the Capsule group compared to the PC and Syringe groups at day 7, before infection (*P* = 0.017). There were no differences (*P* > 0.05) at day 10, after infection. The CRP levels collected after infection were higher than pre-infection concentrations (5.56 vs. 2.20 μg/mL, *P* = 0.035); however, there were no differences (*P* > 0.05) between the treatment groups.

## Discussion

The new delivery method that used a more concentrated dose of ETEC in gelatin capsules resulted in the highest prevalence of ETEC-F4 diarrhea compared to the more usual method of delivering a broth culture by drenching gun. Although the Capsule method delivered the lowest total concentration of ETEC, pigs developed more diarrhea after infection, most likely due to the direct delivery method where the capsules dissolved and the ETEC was released. In this study, approximately 66% of pigs in the PC group did not receive the full inoculum due to spillage and difficulty swallowing the broth. Furthermore, the conventional dosing method (PC) took approximately 4 times longer to administer compared to the Capsule method, which appeared to cause greater stress and led to the removal of 1 pig due to development of respiratory disease associated with inhalation of the ETEC-F4 broth. Direct inoculation of ETEC-F4 into the gastrointestinal tract (GIT) can also be achieved using intragastric gavage, but evidence suggests this method causes significant stress (Sørensen et al., 2009; Sugiharto et al., 2014; Luise et al., 2019). Therefore, using capsules to deliver ETEC-F4 combined with sourcing MUC4+ pigs can reduce the number of animals used in inoculation studies, addressing the 3R’s of replacement, refinement, and reduction, as well as reducing the risk of ETEC-F4 inhalation.

By extension, the DI was significantly higher in pigs dosed using the Capsule method compared to the NC group. The same treatment group also had the lowest standard deviation in DI suggesting that this delivery method minimized infection rate variation. The Capsule group pigs were guaranteed to ingest the correct and same amount of ETEC, as the capsules were swallowed. In contrast, spillage associated with the drenching gun (PC) and the Syringe, albeit the latter at a much lower dose, would have resulted in less ETEC reaching the small intestine to attach, resulting in a greater variation in the incidence of diarrhea and the DI. In a recent review by Luise et al. (2019), the authors suggested genetic susceptibility, immune competence, and previous exposure to *E. coli* are the causative agents for the large experimental variation in ETEC diarrhea. The same review (Luise et al., 2019) found that although the volume of the broth given varies between studies, e.g., 1 mL (Spitzer et al., 2014), 1.5 mL (Trevisi et al., 2015a, 2015b), 6 mL (Molist et al., 2012), and 15 mL (Hedegaard et al., 2016), all doses were approximately the same concentration and administered in the back of the oral cavity using a liquid suspension. The current study demonstrated that the use of a liquid ETEC inoculum can be another causative factor in the variation of ETEC diarrhea, with gelatin capsules delivered per os having the ability to minimize this.

Partially susceptible pigs had higher fecal ETEC-F4 shedding scores than their resistant counterparts. Furthermore, sequencing that was undertaken on the ETEC recovered from the feces following challenge verified that the ETEC shed was the same serotype (O149:K88) as the inoculation culture. These data support the hypothesis that pigs with the F4 receptor will develop more diarrhea and shed more ETEC in feces than pigs without the F4 receptor when experimentally challenged with ETEC-F4. This is supported by the DI data, with the scores being 6 times higher in susceptible pigs when compared to resistant pigs. Although only 56% of partially susceptible pigs in the infection treatment groups developed diarrhea, the results were influenced by the success of the different infection methods and the pigs MUC4/− status. These data are generally consistent with previous research on susceptible pigs developing more diarrhea than resistant pigs (Casini et al., 2003; Jensen et al., 2006; Trevisi et al., 2009; Luise et al., 2019). In a recent study, Sterndale et al. (2019) found that PCR-RFLP is not a reliable indicator for identifying MUC4+/− propensity. This could explain the lower infection rate and indicate that DNA sequencing should be undertaken to identify MUC4+/− propensity. Furthermore, Goetstouwers et al. (2014) found that MUC4 and Mucin 13 are not strongly associated with ETEC susceptibility, and orphan genes located on chromosome 13 have a higher affinity for identifying ETEC-F4 susceptibility in pigs.

Despite the Capsule group developing more diarrhea, no significant differences were established in the blood parameters between the 4 treatment groups. This could suggest pigs were not sufficiently stressed to observe changes in blood markers, analysis was completed on incorrect blood markers, or blood samples were taken too late to coincide with peak infection.

### Conclusion

This study showed that genetically testing pigs for susceptibility and using a Capsule to deliver ETEC can increase cases of ETEC-F4 diarrhea and success of ETEC infection in experimentally challenged pigs. Taken together, these methods have the potential to reduce the number of pigs needed in future experimental infection studies.
